# Characterization of host plant resistance to zebra chip disease from species-derived potato genotypes and the identification of new sources of zebra chip resistance

**DOI:** 10.1371/journal.pone.0183283

**Published:** 2017-08-23

**Authors:** Mahnaz Rashidi, Richard G. Novy, Christopher M. Wallis, Arash Rashed

**Affiliations:** 1 Department of Entomology, Plant Pathology and Nematology, University of Idaho, Aberdeen Research and Extension Center, Aberdeen, Idaho, United States of America; 2 University of Florida, Department of Entomology, Citrus Research and Education Center, Lake Alfred, Florida, United States of America; 3 United States Department of Agriculture, Agricultural Research Service, Small Grains and Potato Germplasm Research Unit, Aberdeen, Idaho, United States of America; 4 United States Department of Agriculture, Agricultural Research Service, Crop Diseases, Pests and Genetics Research Unit, Parlier, California, United States of America; Montana State University Bozeman, UNITED STATES

## Abstract

*‘Candidatus* Liberibacter solanacearum’ (Lso), an uncultivable phloem-limited phytopathogenic bacteria, is known to be associated with Zebra Chip disease (ZC), which represents a major threat to potato production in the US and elsewhere. This pathogen is transmitted by the phloem-feeding potato psyllid, *Bactericera cockerelli* Sulc (Hem. Triozidae). Currently, there are no reports of resistance to ZC in cultivated potatoes. This greenhouse study was conducted to evaluate Lso transmission success and the susceptibility of 11 potato breeding clones, representing diverge genetic background, to ZC, in relation to a susceptible commercial cultivar, Russet Burbank. Individual plants were exposed to two Lso-positive potato psyllids for 48 hours. The percentage of successful Lso transmission varied across the evaluated genotypes ranging between 7 and 57%. Freshly-cut and fried tubers showed significant variation in ZC symptom severity among the breeding clones, with several genotypes expressing relative tolerance when compared to Russet Burbank. None of the evaluated clones showed statistically lower Lso titers than Russet Burbank with the exception of one genotype in the second year of the study. However, the presence of a non-significant relationship between average symptom severity and Lso titer indicated variations in phenotypic responses (i.e. tolerance) to Lso existed among evaluated breeding lines. Breeding clones A07781-3LB, A07781-4LB and A07781-10LB had relatively lower Lso titer (low susceptibility) and tuber symptom expression (high tolerance) among the tested genotypes. As these three clones represent full siblings, the observed effects could be indicative of the presence of a genetic basis for resistance/tolerance to ZC. Findings provide a better understanding of resistance/tolerance to ZC, and contribute to continued efforts in breeding for resistance to this disease.

## Introduction

Zebra chip (ZC) disease has been responsible for millions of dollars of losses in potato (*Solanum tuberosum* L.) producing regions of the United States, as well as, Central America, and New Zealand [1]. In the USA, the disease was first reported in Texas in 2000, and has since been reported in Arizona, California, Colorado, Idaho, Kansas, Nevada, New Mexico, Nebraska, Oregon, Washington, Idaho, and Wyoming [2, 3]. The putative causal agent of ZC, ‘*Candidatus* Liberibacter solanacearum’ (Lso), is a gram negative, and phloem-limited bacterium, which is yet to be isolated *in vitro*. Lso is transmitted by the potato psyllid *Bactericera cockerelli* (Sulc) (Hem. Triozidae) [4, 5, 6]; this insect species has a widespread presence in the Western and Central US [1].

ZC-affected plants show foliar symptoms that include stunting, swollen nodes, aerial tubers and axillary buds, leaf scorch, purpling/chlorosis of young leaves, wilting and eventual plant death [6]. The typical tuber symptom is characterized as brown discoloration of the vascular tissue. Chips and other fried products from the infected tubers have a burnt appearance, a trait that renders processed potato products unsuitable for consumers [7, 6].

Currently, management of ZC relies heavily on frequent insecticide applications to reduce vector numbers, and thus, to limit the spread of Lso. Frequent chemical applications would not only have negative impacts on the environment and human health [8, 3], but can also contribute to development of resistance in vector populations [9]. Therefore, investing into development of environmentally friendly, and relatively more sustainable, management approaches as alternatives to, or in integration with, chemical controls becomes essential; the use of plant resistance has been proposed as an effective and long term approach to manage ZC [3, 10, 11]. However, to date no resistance has been identified in the US and New Zealand commercial potato cultivars [12, 3].

Lso transmission among plants is only possible through feeding by the potato psyllid vectors, and potential mechanisms of resistance to vector-borne infections may be explored at the level of both vector-plant and pathogen-plant interactions [13]. Indeed, variations in the potato psyllid response to species germplasm of *S*. *bulbocastanum*, *S*. *habrochaites*, and *S*. *verrucosum* [14, 15, 16] and to unique breeding clones derived from *Solanum etuberosum* and *S*. *berthaultii* [17, 10], as well as various breeding clones response to Lso [18, 11] have previously been reported.

Hereafter, we use the term ‘ resistance ’ or ‘low susceptibility’ to represent a plant’s ability to limit or (reduce) infection level by a pathogen—in this case Lso. The term ‘ tolerance ’ will be used to reflect the extent to which a plant can maintain a healthy appearance (relatively low ZC symptom expression in tuber tissue) despite Lso infection [19]. Currently, there is no published information on the potential resistance or tolerance of any commercial potato varieties to Lso. Thus, as a continuation of efforts to identify sources of resistance, the present study was aimed at quantifying variations in the degree of relative susceptibility to ZC among several potato genotypes including unique germplasm derived from the species *Solanum etuberosum* and *S*. *berthaultii*. To achieve this goal, our main objectives were set to i) compare Lso transmission success by its potato psyllid vectors among the evaluated genotypes, and ii) classify the evaluated genotypes according to their relative susceptibility to Lso infection and tolerance to ZC.

## Materials and methods

### Insect colony

Lso-infected, *B*. *cockerelli*, colony were originally collected from ZC-affected potato fields (research plots) in Weslaco, TX. No field permits were required for this study, as insects were previously collected by other researchers and maintained for several generations. Prior to our experiments, insects were reared on potato *S*. *tuberosum* L. (var. Russet Burbank) in growth chambers, in 60 x 60 x 60-cm tent shaped bugdorm cages, with temperatures ranging between 21 and 26°C, on a 16:8 photoperiod (L:D). The potato psyllids were of the central haplotype [20]. The biotype of the pathogen was known to be Lso-B [21]. Potato plants were replaced as needed. Although all experimental psyllids were eventually tested, 10 adult psyllids were removed from Lso-infected colony, prior to every inoculation event, and tested individually for the presence of Lso, using quantitative polymerase chain reaction (qPCR) (described below). The Lso incidence in our colony was determined at 100% prior to both infestation events (10/10 tested positive).

### Plant material

A total of 11 potato breeding clones representing diverse genetic backgrounds, derived from *Solanum* relatives of cultivated potato species were screened under controlled greenhouse conditions with temperatures fluctuating between 18 (night) and 23°C (day), and a 14:10 h photoperiod (Light:Dark). The potato genotypes used in this study are listed in Table 1. The commercial cultivar Russet Burbank, susceptible to ZC [3], was included as the control in the study. All potato lines were provided by the USDA-ARS, Small Grains and Potato Germplasm Research Unit, Aberdeen, Idaho. The potting mix consisted of 20% peatmoss (Sun Gro Horticulture Canada Ltd., Seba Beach, AB, Canada), 70% sand, and 10% of vermiculite (Therm-o-Rock West INC., Chandler, AZ, USA) and fertilizer (Osmocote; Scott-Sierra Horticultural Products Co., Marysville, OH, USA). Potato plants were grown in 2-gallon pots. In each of the two years of evaluations, there were twelve plant replicates per genotype, including two non-infested controls.

**Table 1:** Potato breeding clones and their background used in this study.

Clone	Background / selection basis
A05214-3LB	Putative ZC resistance, cultivar Defender as grandparent
A07781-10LB, A07781-3LB, A07781-4LB (unpublished data)	**Derived from *Solanum* species *S*. *chacoense***. The three clones represent full sibs from family A07781; other siblings previously identified as ZC resistant in OR & TX field screening, with preliminary evidence for putative resistance to Lso.
463–4, P2-4, A05379-211, A07701-8LB, A07705-4LB, A07701-6LB (Butler et al. 2011, Diaz-Montano et al. 2013)	**Derived from solanum species *S*. *etuberosum* and *S*. *berthaultii*.** Potential impact on vector fitness and Lso transmission, clones represent haploid x *S*. *berthaultii* parent (463–4), BC_1_ (P2-4) and BC_4_ (4 remaining clones)
A02449-100	Putative resistance to ZC
Russet Burbank (Munyaneza et al. 2011)	Susceptible control; US commercial cultivar

### Plant inoculation, and tuber evaluations

Inoculations were conducted on a single fully developed leaflet of germplasm entries approximately 3 weeks following planting, with the release of two Lso-positive potato psyllids into a 1-inch clip cage (BioQuip, Rancho Dominguez, CA), an infestation level which could frequently occur under natural circumstances. Two plants per genotype were not infested and were included as non-infected controls. Plants, including the non-infested controls, were covered individually with large mesh bags for the 48-hour inoculation access period (IAP), to prevent any potential escapes into the greenhouse. The two potato psyllids were then collected from each plant, placed into a 2 ml tube, and stored at -20°C for DNA extraction (composite sample of the two psyllids) and qPCR analysis. Plants were sprayed with m-pede insecticidal soap on day 7, and with Warrior II on day 14, post- inoculation, to assure removal of any potential nymphs that might have hatched from eggs laid during the IAP. The inoculated plants were maintained in the greenhouse and tubers were harvested approximately 7–8 weeks after the onset of vine senescence. Tubers less than 2-cm in length were excluded from the study. Each tuber was then sliced at the stolen attachment end and scored for visual symptoms of ZC based on a 0 to 3 scale [22], with ‘0’ representing asymptomatic tubers and ‘3’ representing tubers with sever discoloration symptoms. A 6-mm Harris Uni- Core tissue sampler (Ted Pella, Inc., Redding, CA) was used to remove core samples, from the stolon attachment ends, for later Lso quantifications. Then each sampled tuber was cut in half from stem to bud end and two chips were sliced from the middle of each tuber. The potato slices were rinsed using tap water to remove starch and were placed on a paper towel to remove water. The slices were fried in vegetable oil heated to 177°C for approximately 5 min. Each chip sample was scored for ZC symptoms using scale 0–3 [9] where 0 = no discoloration, and 3 = severe dark brown discoloration.

### DNA extraction of psyllid and potato tuber

Psyllid adults were ground in a homogenizer for DNA extraction (described below). Total DNA of psyllid adults (composite sample of two psyllids/plant) was extracted according to Marzachi et al. [23]. The DNA was eluted in 50 μl of double-distilled water (ddH2O) and stored at -20°C. Overall, 96.82% of the psyllids samples tested positive. Plants with no positive psyllids were excluded from the dataset.

Total DNA was extracted from healthy and ZC-infected potato tissue. 100 mg of potato tuber tissue, in a 2-ml microcentrifuge tube, was placed immediately in liquid nitrogen, following removal from -80°C. Tissue was ground in a homogenizer (Omni International Inc., Kennesaw, GA) for 2 min, using 2.8 mm ceramic 325 g beads, at high speed. Then, 200 μl of lysozyme stock solution (10 mg/ml lysozyme in 10 mM Tris-HCl pH 8, Sigma-Aldrich, Bellefonte, PA) was added and the mix was incubated at 37°C for 30 min. After incubation, 800 μl of CTAB (100 mM Tris-HCl pH 8, 20 mM EDTA pH 8, 1.4 M NaCl, 2% CTAB, 2% PVP, and 0.2% β-Mercaptoethanol) was added to the homogenate, and the sample was incubated for 30 min at 65°C. Following incubation, an equal volume of chloroform:isoamylalcohol (24:1) was added. Samples were vortexed and then centrifuged at 14,000 rpm for 15 min. The aqueous layer was then transferred to the new microfuge and 1 volume cold isopropanol and 0.1 volume NaCl 5 M were added. The tubes were placed in -20°C overnight. DNA was precipitated at 14,000 rpm for 20 min at 4°C. The pellet was washed with ice-cold 70% ethanol and centrifuged at 14,000 rpm for 2 min. The DNA pellet, after air-drying, was dissolved in 50 μl of double-distilled water (ddH2O).

### Quantification of Lso titer in psyllid and potato tuber by qPCR

Absolute quantification method was used to quantify Lso copy numbers in both the potato psyllid vector and potato tuber tissue. The qPCR was performed with 15 μl of reactions containing TaqMan® Environmental Master Mix 2.0 (Life Technologies, Carlsbad, CA), 400 nM primer LsoF (5ʹ-GTC GAG CGC TTA TTT TTA ATA GGA-3ʹ; [24]), 400 nM primer HLBr (5 ʹ-GCG TTA TCC CGT AGA AAA AGG TAG-3ʹ; [25]), 200 nM probe HLBp (6FAM 5 ʹ - AGA CGG GTG AGT AAC GCG -3', TAMRA, Life Technologies, Carlsbad, CA; [25]), and 1 μl of DNA template. Both forward and reverse primers were synthetized by the Integrated DNA Technologies, Inc., Skokie, IL. The real time PCR program consisted of 1 cycle at 95°C for 10 min followed by 40 cycles of 95°C for 15 s, and 60°C for 1 min. Reactions were performed in a CFX Connect Real Time System (Bio Rad, Hercules, CA, USA). A positive control (DNA of Lso positive), a negative control (DNA of healthy tuber), and water control (no template control, NTC) were included in all qPCR.

### Statistical analysis

A Generalized Linear Mixed Model (GLMM), with a logit link function assuming a binomial distribution, was used to examine whether the probability of successful transmission can be predicted by host plant genotype. Four randomly selected tubers per plant were tested for Lso presence (S1 Table). A plant was considered infected where, at least, one of the sampled tubers tested positive for Lso. Genotype was treated as the fixed factor. Year was treated as the random factor. Lso titer in psyllid, hereafter referred to as ‘infection level’, was included as a continuous covariate in the initial model. This covariate was later removed from the final model due to its non-significant effect (*P* > 0.05).

Fresh and fried symptom severities of the tuber tissue were compared among the experimental genotypes, using GLMM. A multinomial distribution was assumed and a logit link function was applied. The analyzed symptom data was based on average of 2 tubers per plant in 2014 and 4 tubers per plant in 2015. Plants that tested negative for the pathogen, based on tuber analyses, were excluded from symptom severity data to avoid bias (but see below). Potato genotype, year (random) and the interaction between the two were the factors included in the initial model. The interaction term was not included in the final models due to the non-significant effect (*P* > 0.05).

Spearman rank correlation (ρ) was used to evaluate correlation between fried and fresh symptom severities, as well as between the Lso titer within tubers of each genotype and average symptom severity in fresh tuber tissue.

GLMM was also used to compare Lso quantities in tuber tissues calculated based on average of 2 randomly selected tubers per plant in 2014 (same tubers scored for symptoms) and 4 tubers per plant in 2015. Plants that tested negative, based on the four evaluated tubers, were excluded to avoid potential bias. Log-transformed Lso was normally distributed and an identity link function was applied. Potato genotype, year and the two-way interaction term were included in the initial model. Year was treated as a random factor. The two breeding clones A07781-3LB and A07781-10LB were excluded from both tuber symptom and Lso titer analyses due to insufficient numbers across both years, which could, however, be indicative of resistance to Lso and/or insect feeding in these clones. Least Significant Difference (LSD) was used for pairwise comparisons. Fresh and fried symptom and Lso data are presented in S2 Table.

**Fig 1:**
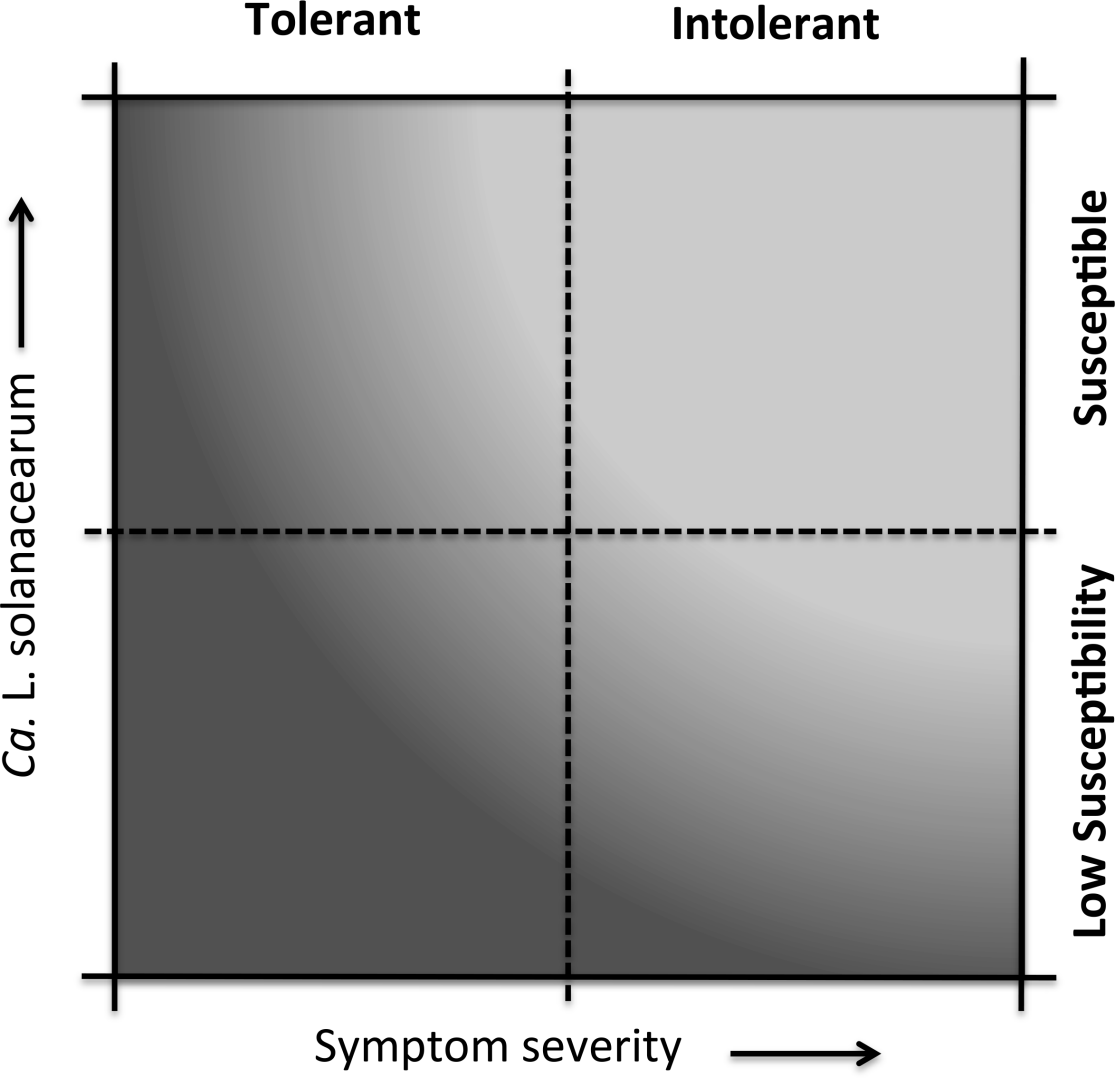
Schematic figure, illustrating classification based on relative susceptibility and tolerance. Dotted lines represent overall averages of the experimental genotypes. The darkest gradient reflects low susceptibility and high tolerance.

To assess the relative susceptibility/tolerance among breeding clones we placed individual genotypes, with respect to their calculated average Lso titer and average symptom score, into a scatter plot graph (Y = Lso titer, X = symptom score) divided into four quadrants based on average symptom score and average Lso titer across all evaluated genotypes [26, 27] (Fig 1). This relative classification was conducted both including the infested, yet Lso-negative plants, and excluding those plants that tested negative. While including Lso-negative plants may potentially impose bias on final conclusion, it would help to account for genotypes, which might have had some defense mechanism to contain Lso infection soon after inoculation by the vector. Based on these categorizations, potato genotypes with lower than average Lso titers were considered relatively less susceptible (resistant) and those with lower than average tuber symptom severity were considered as relatively more tolerant (low symptom expression). Potato genotypes with greater than average tuber symptom severity and greater than average Lso titer were classified into intolerant and susceptible quadrants, respectively (Fig 1).

Since classifications based on relative susceptibility and tolerance were primarily descriptive, they were followed by hierarchical cluster analysis to provide an objective examination for patterns of clustering among genotypes. Average Lso titer and symptom severity for each of the genotypes was used in this classification analysis. Between-groups linkage method and squared Euclidean distance measures were employed.

All analyses were carried out in IBM-SPSS, version 22 (IBM, Armonk, NY, USA).

## Results

### Lso transmission success in greenhouse inoculations

**Fig 2:**
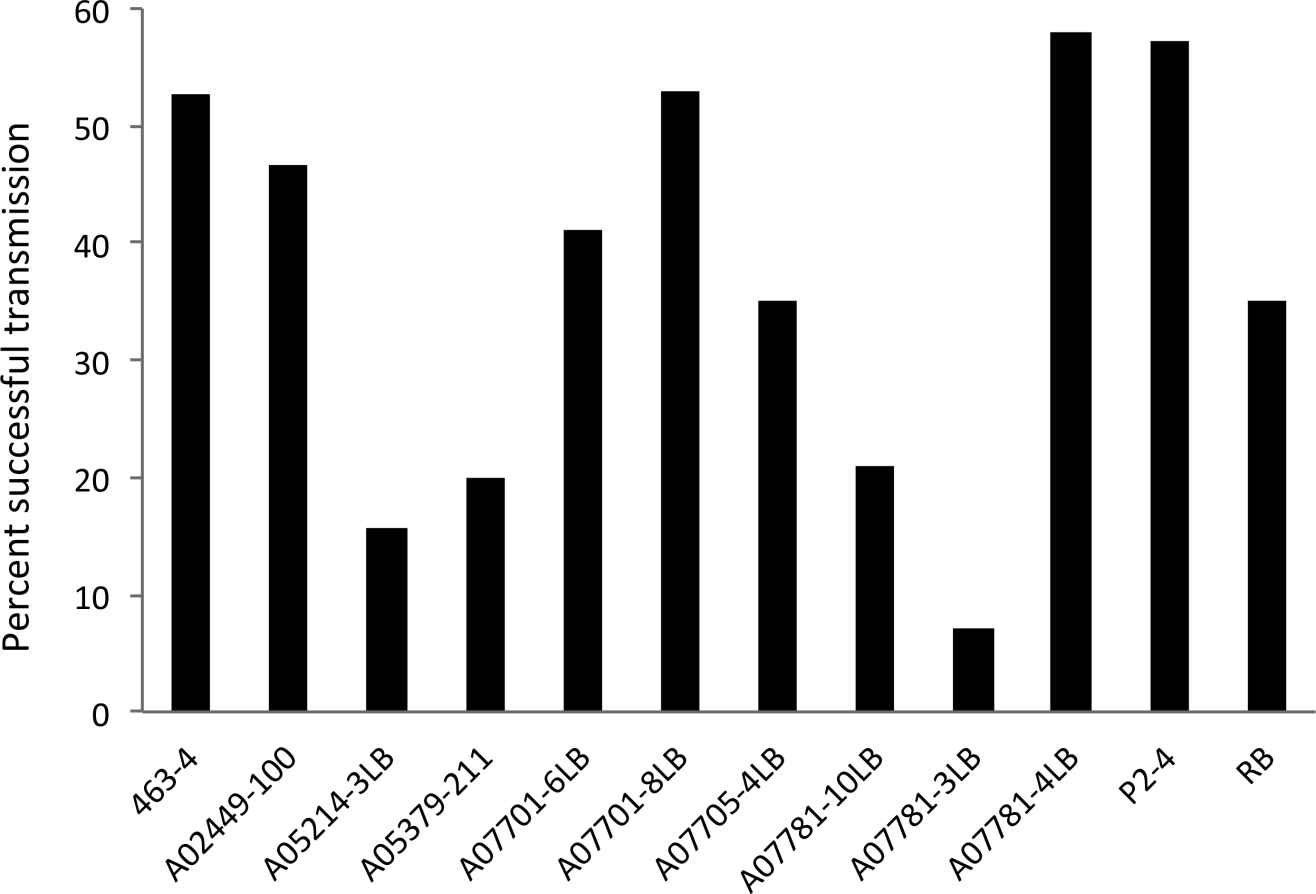
Percentage of plants of each clonal entry, infected with ‘*Candidatus* Liberibacter solanacearum’, following plant inoculation with Lso-positive psyllids. Values reflect an average over two years.

The probability of successful transmission varied significantly among genotypes (GLMM; F _*11*,*208*_ = 2.01; *P* = 0.029) with infection rates between 7% (A07781-3LB) and 58% (A07781-4LB) (Fig 2). Despite the observed range in rate of transmission, the evaluated breeding clones did not differ significantly from that of the susceptible control, Russet Burbank (all *Ps* > 0.092). The average transmission of Lso across all evaluated genotypes was calculated at 36.9%, with Russet Burbank (35%) and A07705- 4LB (35%) being near the average rate. A05379-211, A07781-3LB, A07781-10LB and A05214-3LB had transmission rates below the average, with A07781-3LB having the lowest percentage of infected plants at 7%. Transmission success was relatively higher, and greater than the overall 36.9% average, in the remaining 6 genotypes (Fig 2). The infection level of the potato psyllids was not a predictor of Lso transmission success (F _*1*,*188*_ = 0.623, *P* = 0.431).

### Zebra chip symptom severity

**Fig 3:**
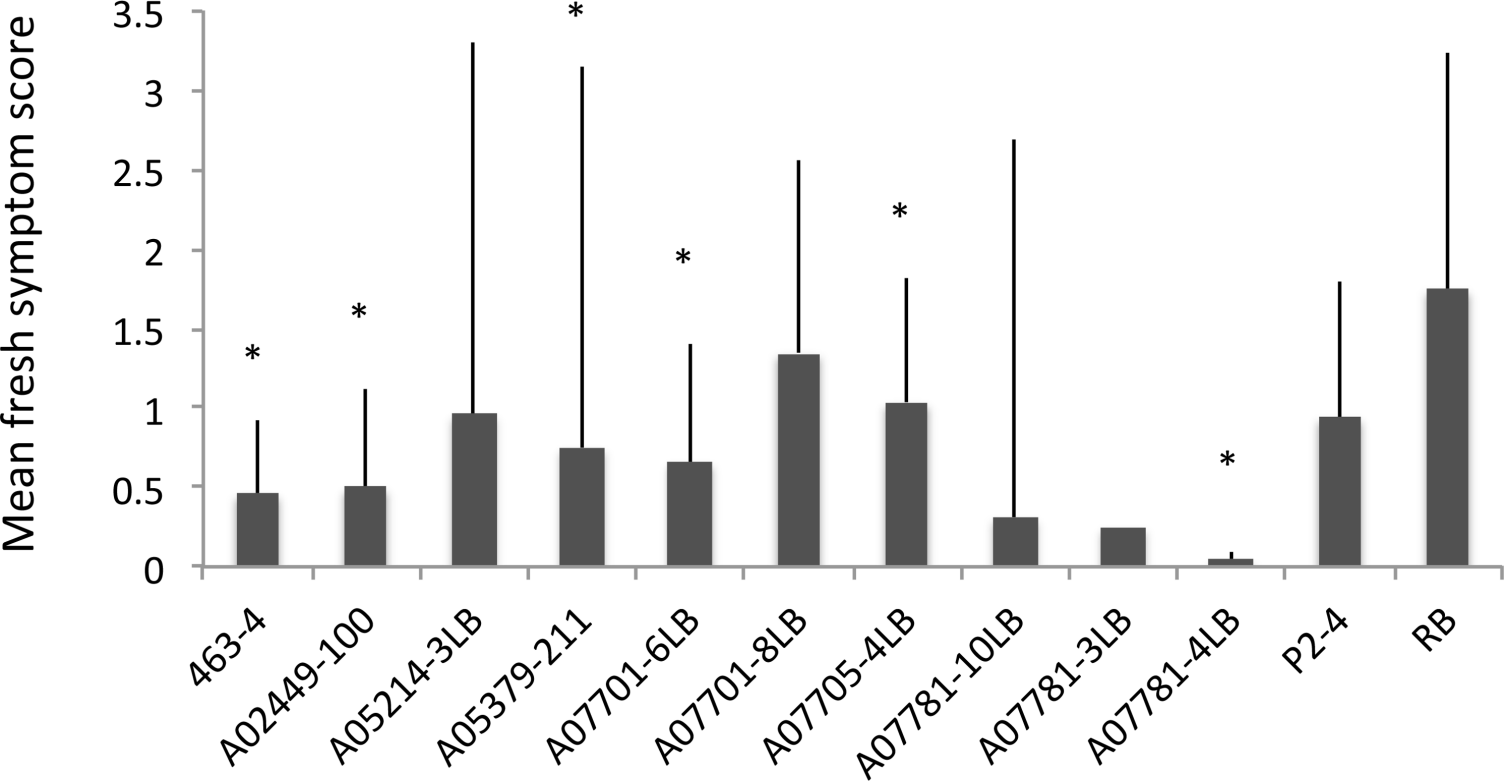
Mean fresh tuber visual ZC symptom severity score (0–3) in different potato germplasm. With the exception of A07781-3LB and A07781-10LB, which were excluded from statistical analysis due to insufficient number of infected plants, significant differences between the evaluated breeding lines and Russet Burbank, are marked with asterisks. Error bars represent 95% confidence intervals.

Zebra Chip symptom severities of the freshly-cut tubers varied significantly among the evaluated cultivars as revealed by GLMM (F _*9*,*52*_ = 2.37; *P* = 0.025; Fig 3); average symptom scores were significantly lower in entries 463–4, A02449-100, A05379-211, A07701-6LB, A07705-4LB and A07781-4LB than the susceptible control, Russet Burbank. A07781-4LB was the genotype expressing the least symptom severity consistently in both years of the study. Although A07781-3LB and A07781-10LB were not included in statistical comparisons, both genotypes expressed relatively low ZC symptoms. No significant interaction was detected between year and genotype (F _*9*,*43*_ = 1.68, *P* = 0.124).

**Fig 4:**
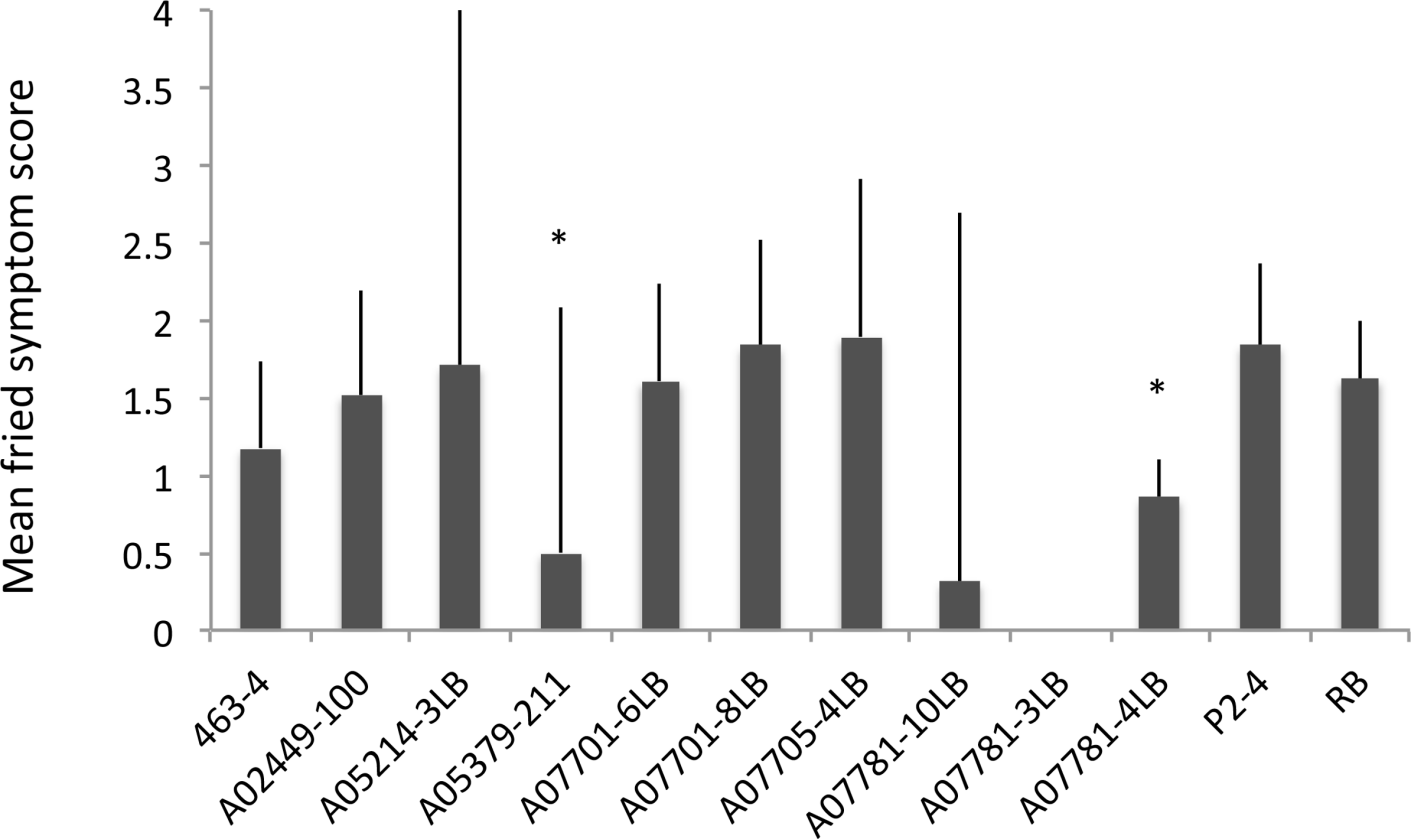
Mean visual ZC symptom severity on fried slices scored (0–3) in different potato genotypes. Significant differences from the susceptible control, Russet Burbank, are marked with asterisks. Error bars represent 95% confidence intervals.

The significant variation in symptom severities also persisted in fried products (F _*9*, *51*_ = 3.07, *P* = 0.005; Fig 4). Symptom expression of ZC in chips of A05379-211 (*P* < 0.001) and A07781-4LB (*P* = 0.035) were significantly lower than Russet Burbank. No significant year-by-genotype interaction was detected (F _*9*, *42*_ = 1.06, *P* = 0.409).

**Fig 5:**
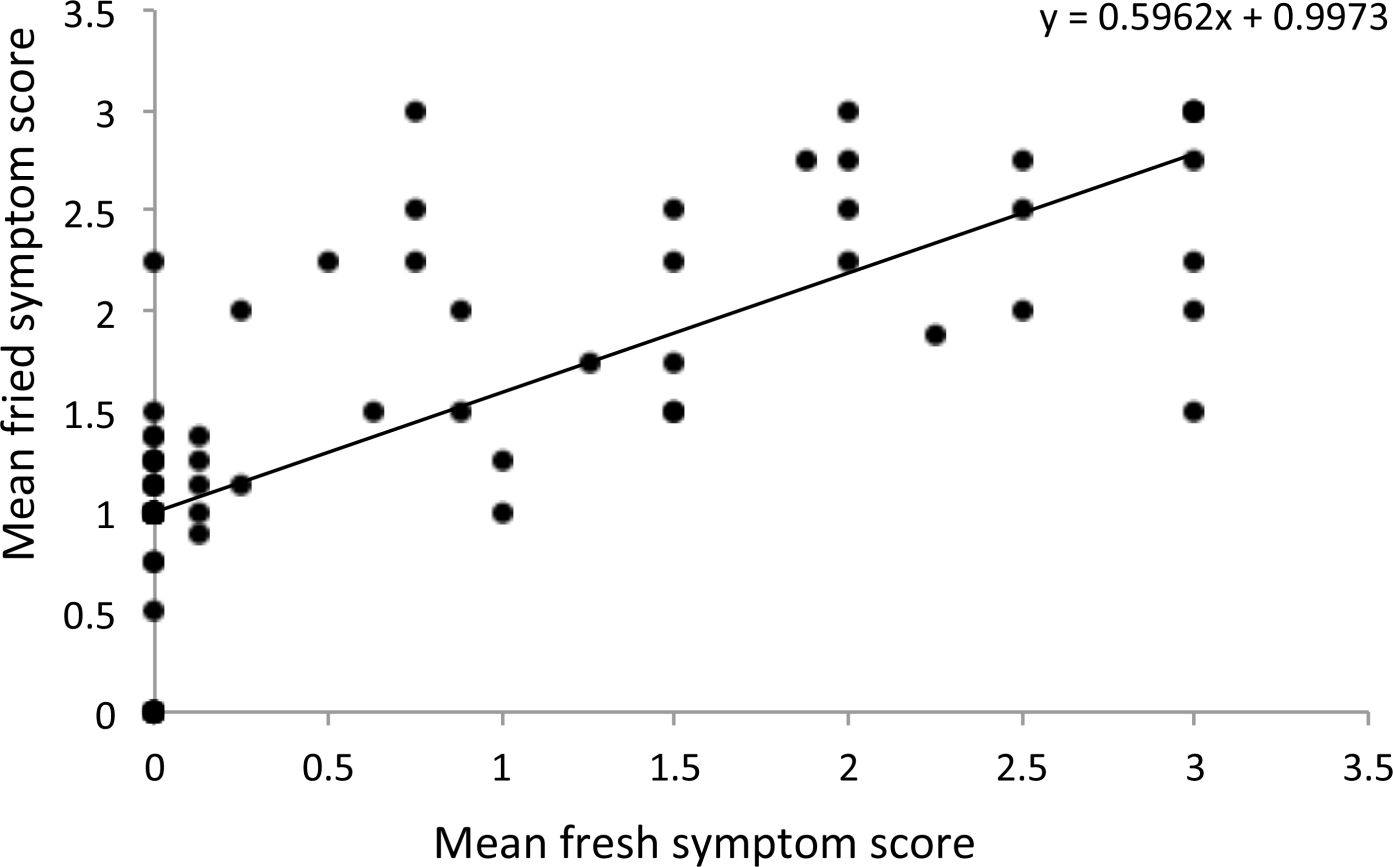
The relationship between zebra chip severity scores of fresh and fried slices.

Overall, a significantly positive correlation was detected between fresh and fried ZC symptom scores (ρ = 0.796, *P* < 0.001; Fig 5).

### Lso quantification in the evaluated potato genotypes

**Fig 6:**
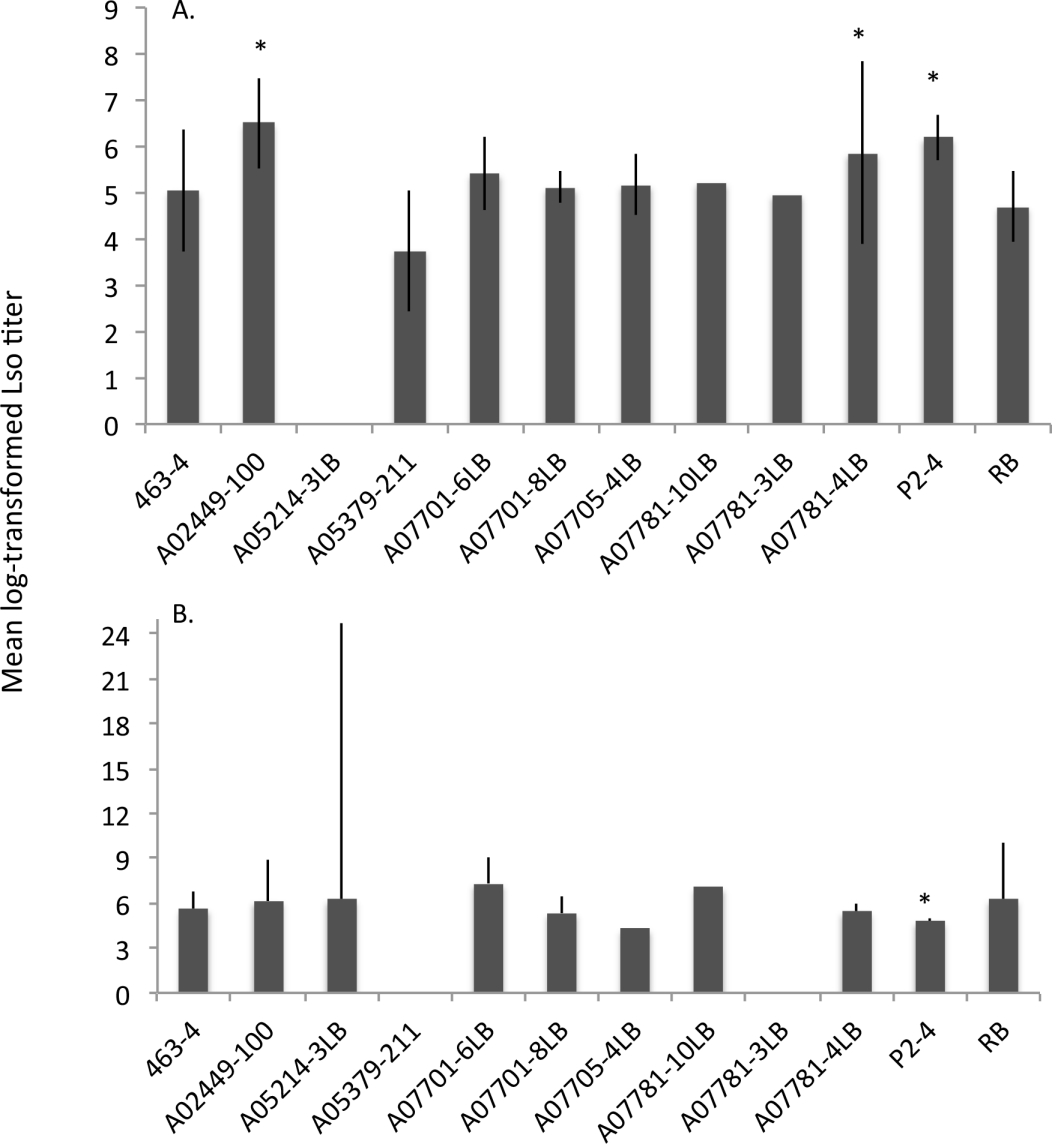
Mean log-transformed ‘*Candidatus* Liberibacter solanacearum’ titer among the evaluated genotypes in 2014 (A), and 2015 (B). A07781-3LB and A07781-10LB were excluded from the 2015 analysis due to an insufficient number of transmission events. Significant differences from the susceptible control, Russet Burbank, are marked with asterisks. Error bars represent 95% confidence intervals.

Lso titer varied significantly among the evaluated genotypes (F _*9*, *56*_ = 2.59; *P* = 0.014). Since a significant year-by-genotype interaction (F _*9*, *56*_ = 2.34; *P* = 0.026), results are presented separately (Fig 6A and 6B). In 2014, A02449-100, A07781-4LB and P2-4 had significantly higher titer levels than Russet Burbank. A05379-211 had the least Lso titer, however, it was not significantly different from the Russet Burbank control. In 2015, P2-4 was the only clone with significantly lower Lso titer than Russet Burbank, an observation that contradicted the 2014 result for this particular genotype.

The proportion of tubers that tested positive from infected plants was not statistically different across genotypes (F _*11*, *282*_ = 0.868; *P* = 0.572). A significant year effect was detected (F _*1*, *282*_ = 12.99; *P* < 0.001). The interaction term was excluded due to largely non-significant effect (*P* = 0.320).

### Relationship between fresh cut tuber ZC symptom severity and Lso titer

**Fig 7:**
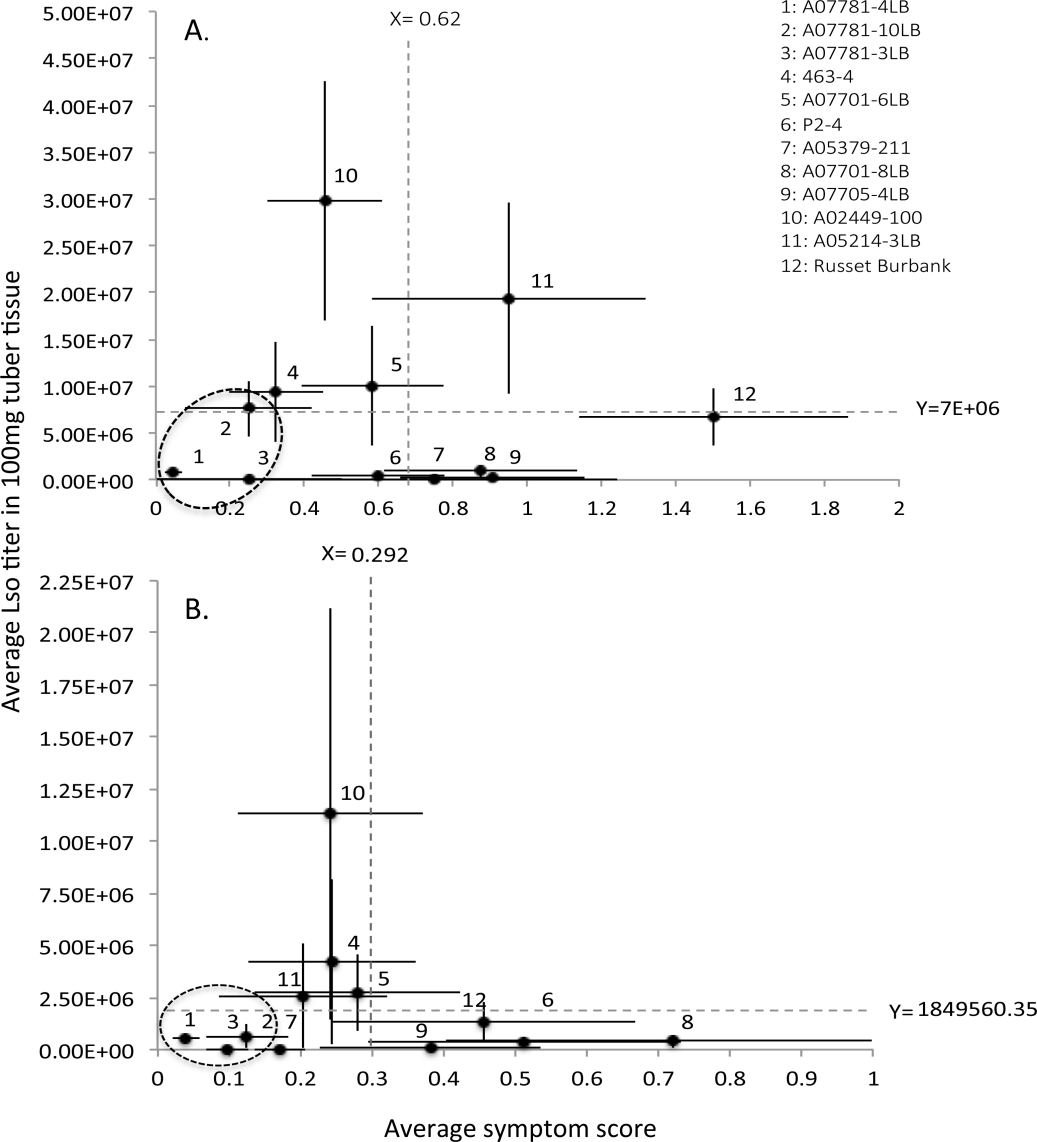
Relationship between zebra chip symptom severities of fresh tuber slices and Lso titer in the evaluated entries, both excluding (A) and including (B) vector-infested plants that tested negative for Lso presence. Error bars represent standard errors (±1).

To assess relative susceptibility (resistance) and tolerance among the 12 evaluated genotypes, the overall relationship between Lso titer and ZC symptom severity in freshly cut tubers, pooled across 2014 and 2015 time-blocks, was examined. This examination was performed on datasets that both included (ρ = 0.091, N = 12, *P* = 0.779) and excluded (ρ = 0.028, N = 12, *P* = 0.931) the infested plants that tested negative for Lso and no significant correlation were detected in either approach. Despite this non-significant relationship, relative susceptibilities and tolerances were as follow: A07781-3LB and A07781-4LB consistantly fell into low susceptibility/tolerant category. Considering only plants that tested positive for Lso, A07781-10LB was classified as borderline resistant and tolerant. Including plants that also tested negative into the analysis moved A07781-10LB into low susceptibility/tolerant category along with A07781-3LB, A07781-4LB and A05379-211. In both approaches, A02449-100 was classified as a susceptible, but tolerant genotype, while Russet Burbank remained in the category representing relatively less susceptible, yet intolerant genotypes (Fig 7A and 7B).

**Fig 8:**
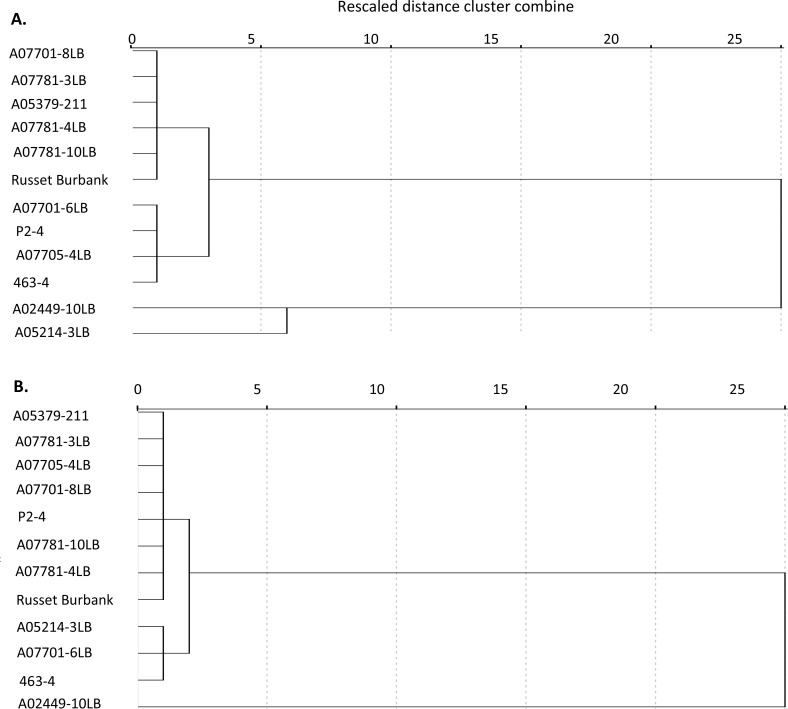
Clustering patterns of the evaluated genotypes based on hierarchical cluster analysis, excluding (A) and including (B) vector-infested plants that tested negative for Lso presence.

In spite of the observed similarities between genotype classifications conducted based on either approach, patterns of clustering showed differences between the two approaches. When non-infected plants were not included, the three A07781 siblings clustered with Russet Burbank, A05379-211 and A07701-8LB. This group neighbored another cluster of 4 breeding clones that included P2-4, A07705-4LB and 463–4. A02449-10LB and A05214-3LB grouped together, distanced from the other two formed groups (Fig 8A).

When the non-infected controls were included in the analysis, the majority of the genotypes clustered together, closely, in a group that also included A07781 siblings as well as Russet Burbank (Fig 8B). The next closest group contained the three breeding clones A05214-3LB, A07701-6LB and 463–4. The single genotype A02449-10LB was placed distanced from the remaining evaluated genotypes (Fig 8B).

## Discussion

Potato host susceptibility and tolerance to zebra chip disease was assessed among 11 potato breeding clones in relation to Russet Burbank, the most widely grown potato cultivar in the U.S. The relative susceptibilities and tolerances were evaluated among genotypes with respect to Lso transmission success, ZC symptom severity in fresh and fried tissues, and Lso titer in tubers.

Challenging the selected genotypes with Lso-carrying potato psyllids, over a two-year study, resulted in Lso transmission rates that ranged between 7 and 58%. However, despite this wide range of variation, no statistically significant differences were detected between either of the 11 breeding clones and Russet Burbank. Potato psyllids are known to be efficient vectors of Lso with a single potato psyllid having an average transmission success of 46% [28]. Likewise, an earlier study Butler et al. [17] reported transmission success rates ranging between 30 and 100% (averaged at 50.9%) among the genotypes included in their study. The 100% transmission success observed in some genotypes, may have resulted from exposing the plants to 10 infective potato psyllids for inoculation purpose; it is known that the likelihood of successful Lso transmission increases with the number of potato psyllid vectors [28, 29]. Such high infestation levels (i.e. 10 psyllids/plant) however, are less likely to occur under natural circumstances as the threshold for insecticide applications are far below these numbers.

Overall, the lowest Lso incidence was observed in A07781-3LB, which was followed by the entries A05214-3LB, A05379-211, and A07781-10LB. As A07781-3LB and A07781-10LB are related full-siblings (family A07781), this might suggest the presence of underlying genetically linked resistance mechanism(s) to transmission of Lso. Apparent segregation in the rate of successful transmission in this family, however, was observed with full-sibling A07781-4LB having the highest percentage of infected plants at 58%. The observed rate of successful transmission for 463–4 was 52%, closely aligned with a 50% transmission success previously reported for this breeding clone by Butler et al. [17]. However, we observed a lower rate of successful transmission in A05379-211 (20%) than the 44% reported by Butler et al. [17]. As mentioned earlier, variability in transmission rates may, at least in part, relate to differences in the number of infective psyllids used between the two studies. This observation, however, was not consistent across all genotypes, suggesting potential differences among plant genotypes in response to increase in the vector numbers.

In addition to their relatively lower transmission success rate, A07781-10 LB and A07781-3LB were among varieties expressing the lowest ZC severities in fresh-cut tubers (Fig 3). However, these two breeding clones were not included in the statistical analyses due to insufficient numbers. Further replicated studies focused on these two genotypes are warranted to objectively confirm the observed trend. Nonetheless, low transmission success and Lso quantities might be indicative of low susceptibility of these entries to Lso and/or the psyllid vector. Of note was full sibling A07781-4LB, which although having the highest Lso transmission rate, it still exhibited the lowest ZC symptom expression in its fresh-cut tubers, indicating tolerance to ZC symptoms (Fig 3).

The three full siblings of family A07781 were also amongst the lowest for ZC tuber symptom severity following frying along with A05379-211 (Fig 4). With A07781-3LB and A07781-10LB excluded from statistical analyses, pairwise LSD only detected significant differences between A07781-4LB and A05379-211, with the susceptible control, Russet Burbank.

The three A07781 full siblings also had the lowest Lso titers, with A07781-3LB and A07781-4LB categorized in the less susceptible/tolerant category, whereas A07781-10LB was categorized as a borderline susceptible, but tolerant, genotype when only plants that tested positive for Lso were used for relative susceptibility/tolerance classification (Fig 7A). Including plants, which were infested but tested negative for Lso, in the relative classification graph, moved all A07781 full siblings into the low susceptibility/tolerant category (Fig 7B). These three full siblings are derived from *Solanum* species *S*. *chacoense* suggesting the possibility of a common source of resistance, and providing support for the assertion that sources of tolerance, and resistance, to Lso may be present in wild species of potato [11, 15].

Russet Burbank exhibited the greatest tuber symptom severity (highly intolerant) despite its relatively low Lso titer (low susceptibility). Indeed, Russet Burbank, among several other commercial varieties, has been reported as a highly susceptible to ZC [3]. Several breeding clones expressed significantly lower symptom severities in fresh-cut tubers than Russet Burbank (Fig 3), with the severity of ZC symptoms also being significantly lower in fried slices of A05379-211 and A07781-4LB (Fig 4). While the low transmission rate of Lso in A07781-3LB and A07781-10LB precluded their inclusion in statistical analyses of ZC tuber symptoms, the lowered transmission rate effectively contributed to reduced ZC tuber symptom expression. A05214-3LB was the only breeding clone that was ranked both susceptible and intolerant when compared to the other entries. However, A05214-3LB was reclassified into the susceptible/tolerant category when infested plants, including those that tested negative for Lso, were included in the calculations. A07701-8LB, A07705-4LB and Russet Burbank were consistently classified in less susceptible/intolerant category. No relationship was detected between Lso titer and symptom severity among cultivars a finding which suggests the existence of phenotypic diversity in response to Lso infection. Here, we would like to re-emphasize that the terms ‘susceptible’ and ‘tolerant’ are both relative, reflecting rankings within the evaluated pool of the 12 genotypes.

Although hierarchical cluster analysis resulted in formation of distinct homogeneous groups, separations appeared to be primarily corresponding to variations in the degree of susceptibility to Lso (Lso titer). Nonetheless, the three A07781 full siblings clustered together, while the highly susceptible A02449-10LB was placed separately, distanced from the majority of entries (Fig 8A and 8B).

Although experiments were conducted under greenhouse conditions, significant year-by-genotype effect was detected when analyzing Lso titer. The breeding clone P2-4 exhibited the most striking inconsistency as it had significantly higher Lso titer than Russet Burbank, in 2014, rendering this genotype highly susceptible. In 2015, however, Lso titer was significantly lower in P2-4 than the Russet Burbank control. The year effect could have been the consequence of a powdery mildew spread, around flowering time, in the 2015 trial. As host-mediated effects of pathogens can impact vector behavior [30, 31], Lso transmission success [32], as well as infections by other pathogens through systemic acquired resistance (SAR) [33, 34], infection by the powdery mildew could have influenced the interactions among vector, host, and Lso, explaining some of the observed inconsistencies for certain clones.

Among other environmental factors which could affect psyllid feeding rate and behavior, one might argue that variations in vector infectivity and titer load between 2014 and 2015 might have been an influential factor affecting variations in the study outcomes. The infective potato psyllids used for inoculations were tested following the experiments and nearly all (96.8%) were positive for Lso. Thus, it is unlikely that the observed between-year differences were due to variability in psyllid infectivity. Moreover, our analysis detected no relationship between Lso titer load of the potato psyllids and the likelihood of successful transmission, supporting the previous findings in ZC [28].

While our approach was sensitive enough to detect among-genotype variations in the probability of successful transmission, ZC symptom severity and Lso titer, A07781-3LB and A07781-10LB had to be excluded from some of the tuber sample analyses due to small sample sizes reflective of the low transmission rates of Lso in these two clones. While the observed low rate of successful transmission of Lso may be indicative of potential resistance to psyllid feeding, and/or the initially injected inoculum, further detailed studies using greater replications of these two clones is now warranted to better understand the potential mechanism(s) involved. Moreover, as Liberibacter spp. pathogenicity is known to be influenced by environmental factors [35, 36], future field studies are needed to evaluate the potential impact of environmental variability on ZC development in each of the evaluated genotypes.

The third tested sibling of the A07781 family, A07781-4LB did not show reduced infection by Lso, but nonetheless displayed mild symptoms of ZC in fresh and fried tubers, suggesting the presence of possible tolerance. Clone A05379-211 with its relatively lower Lso transmission success, Lso titer and ZC symptom expression in both fresh and fried tubers, also is a promising source of resistance/tolerance to ZC. This clone, derived from *S*. *etuberosum* and *S*. *berthaultii*, also represent unique germplasm that can be hybridized with the divergent *S*. *chacoense*-derived A07781 clones to pyramid ZC resistance/tolerance from diverse genetic backgrounds. Future studies focused on this germplasm, and perhaps other genotypes derived from *S*. *chacoense*, *S*. *etuberosum*, and *S*. *berthaultii*, appear to be promising candidates for breeding programs to utilize in the development of potato cultivars with reduced susceptibility to ZC.

## Supporting information

S1 Table Dataset used for comparing transmission success among the 12 evaluated genotypes. (PDF)

S2 Table Dataset used for comparing fresh and fried zc symptom severities and Lso titer comparisons among evaluated genotypes. (PDF)
