# Supplementary material for: Characterization of host plant resistance to zebra chip disease from species-derived potato genotypes and the identification of new sources of zebra chip resistance
Source: PLoS One. 2017 Aug 23;12(8):e0183283. doi: 10.1371/journal.pone.0183283 (PMC5568414; doi:10.1371/journal.pone.0183283)
Supplement: S1 Table — (PDF) [file pone.0183283.s001.pdf]

Subject	year	Plant	Genotype	Lso status*	Psyllid titer
1	2014	1	A05379-211	1	2350000
2	2014	2	A05379-211	0	883000
3	2014	3	A05379-211	0	37500000
4	2014	4	A05379-211	1	8520000
5	2014	5	A05379-211	1	6740000
6	2014	6	A05379-211	0	2300000
7	2014	7	A05379-211	1	10400000
8	2014	8	A05379-211	0	6340000
9	2014	9	A05379-211	0	130000000
10	2014	10	A05379-211	0	11000000
11	2014	1	A07781-3LB	0	1130000000
12	2014	2	A07781-3LB	0	10700000
13	2014	3	A07781-3LB	1	508000000
14	2014	4	A07781-3LB	0	416000
15	2014	1	A07781-10LB	0	116000000
16	2014	2	A07781-10LB	0	15400000
17	2014	3	A07781-10LB	1	763000000
18	2014	4	A07781-10LB	0	473000000
19	2014	5	A07781-10LB	0	20100000
20	2014	6	A07781-10LB	1	4400000
21	2014	7	A07781-10LB	1	1050000000
22	2014	8	A07781-10LB	0	212000000
23	2014	9	A07781-10LB	0	260000000
24	2014	10	A07781-10LB	0	8670000000
25	2014	1	A07701-6LB	0	3960000000
26	2014	2	A07701-6LB	0	119000
27	2014	3	A07701-6LB	1	10600000
28	2014	4	A07701-6LB	1	386000000
29	2014	5	A07701-6LB	1	1670000
30	2014	6	A07701-6LB	0	753000000
31	2014	7	A07701-6LB	1	26600000
32	2014	8	A07701-6LB	1	7200000
33	2014	1	A07705-4LB	1	54100000
34	2014	2	A07705-4LB	0	60300000
35	2014	3	A07705-4LB	1	28400000
36	2014	4	A07705-4LB	0	18300000
37	2014	5	A07705-4LB	0	844000
38	2014	6	A07705-4LB	1	43100000
39	2014	7	A07705-4LB	1	73300000
40	2014	8	A07705-4LB	1	3450000000
41	2014	9	A07705-4LB	1	

42	2014	10	A07705-4LB	0	
43	2014	1	RB	1	79100
44	2014	2	RB	1	44800000
45	2014	3	RB	1	567000
46	2014	4	RB	0	30500
47	2014	5	RB	0	1940000000
48	2014	6	RB	0	41500000
49	2014	7	RB	1	7940000
50	2014	8	RB	0	2350000000
51	2014	9	RB	0	11000000
52	2014	1	A07701-8LB	0	347000
53	2014	2	A07701-8LB	1	10200000
54	2014	3	A07701-8LB	1	18600000
55	2014	4	A07701-8LB	1	72400000
56	2014	5	A07701-8LB	1	20100000
57	2014	6	A07701-8LB	0	3010000
58	2014	7	A07701-8LB	0	151000000
59	2014	1	A02449-100	0	1610000000
60	2014	2	A02449-100	1	71600000
61	2014	3	A02449-100	0	889000000
62	2014	4	A02449-100	1	742000000
63	2014	5	A02449-100	1	20300000
64	2014	1	A05214-3LB	1	78300
65	2014	2	A05214-3LB	0	65500000
66	2014	3	A05214-3LB	0	6780000
67	2014	4	A05214-3LB	0	1190000000
68	2014	5	A05214-3LB	0	61300
69	2014	6	A05214-3LB	0	1740000
70	2014	7	A05214-3LB	0	3630000
71	2014	8	A05214-3LB	0	5240000
72	2014	9	A05214-3LB	0	124000
73	2014	1	A07781-4LB	1	23900000
74	2014	2	A07781-4LB	0	47700000
75	2014	3	A07781-4LB	0	19600000
76	2014	4	A07781-4LB	1	5380000
77	2014	5	A07781-4LB	0	164000
78	2014	6	A07781-4LB	0	83100000
79	2014	7	A07781-4LB	0	10600000
80	2014	8	A07781-4LB	0	199000
81	2014	9	A07781-4LB	0	1850000
82	2014	1	463-4	0	40800000
83	2014	2	463-4	1	66000000

84	2014	3	463-4	1	56800000
85	2014	4	463-4	0	216000000
86	2014	5	463-4	1	37900000
87	2014	6	463-4	0	245000000
88	2014	7	463-4	0	273000
89	2014	8	463-4	0	3660000
90	2014	9	463-4	0	2710000
91	2014	1	P2-4	1	231000000
92	2014	2	P2-4	0	29100000
93	2014	3	P2-4	1	207000000
94	2014	4	P2-4	0	71800000
95	2014	5	P2-4	0	20000000
96	2014	6	P2-4	1	32800000
97	2014	7	P2-4	0	176000000
98	2014	8	P2-4	1	347000000
99	2014	9	P2-4	1	109000000
100	2014	10	P2-4	0	298000000
101	2014	11	P2-4	0	206000000
102	2015	1	A05379-211	0	123000000
103	2015	2	A05379-211	0	1360000000
104	2015	3	A05379-211	0	163000000
105	2015	4	A05379-211	0	195000000
106	2015	5	A05379-211	0	
107	2015	6	A05379-211	0	41200000
108	2015	7	A05379-211	0	1100000000
109	2015	8	A05379-211	0	495000000
110	2015	9	A05379-211	0	292000000
111	2015	10	A05379-211	0	59100000
112	2015	1	A07781-3LB	0	255000000
113	2015	2	A07781-3LB	0	606000000
114	2015	3	A07781-3LB	0	1530000000
115	2015	4	A07781-3LB	0	43000000
116	2015	5	A07781-3LB	0	89600000
117	2015	6	A07781-3LB	0	985000000
118	2015	7	A07781-3LB	0	2790000000
119	2015	8	A07781-3LB	0	8500000000
120	2015	9	A07781-3LB	0	273000000
121	2015	10	A07781-3LB	0	516000000
122	2015	1	A07781-10LB	0	1090000000
123	2015	2	A07781-10LB	0	2190000000
124	2015	3	A07781-10LB	0	397000000
125	2015	4	A07781-10LB	0	2030000000

126	2015	5	A07781-10LB	0	413000000
127	2015	6	A07781-10LB	1	104000000
128	2015	7	A07781-10LB	0	223000000
129	2015	8	A07781-10LB	0	727000000
130	2015	9	A07781-10LB	0	3160000000
131	2015	1	A07701-6LB	0	1420000000
132	2015	2	A07701-6LB	0	368000000
133	2015	3	A07701-6LB	0	327000000
134	2015	4	A07701-6LB	0	1170000000
135	2015	5	A07701-6LB	0	2130000000
136	2015	6	A07701-6LB	0	
137	2015	7	A07701-6LB	1	2420000000
138	2015	8	A07701-6LB	0	215000000
139	2015	9	A07701-6LB	1	541000000
140	2015	1	A07705-4LB	0	85200000
141	2015	2	A07705-4LB	0	1740000000
142	2015	3	A07705-4LB	0	2050000000
143	2015	4	A07705-4LB	0	717000000
144	2015	5	A07705-4LB	0	1530000000
145	2015	6	A07705-4LB	0	41100000
146	2015	7	A07705-4LB	0	4380000000
147	2015	8	A07705-4LB	1	1720000000
148	2015	9	A07705-4LB	0	108000000
149	2015	10	A07705-4LB	0	151000000
150	2015	1	RB	0	198000000
151	2015	2	RB	0	482000000
152	2015	3	RB	1	1740000000
153	2015	4	RB	0	149000000
154	2015	5	RB	0	105000000
155	2015	6	RB	1	
156	2015	7	RB	1	385000000
157	2015	8	RB	0	
158	2015	9	RB	0	161000000
159	2015	10	RB	0	247000000
160	2015	11	RB	0	
161	2015	1	A07701-8LB	0	216000000
162	2015	2	A07701-8LB	0	296000000
163	2015	3	A07701-8LB	1	1120000000
164	2015	4	A07701-8LB	1	699000000
165	2015	5	A07701-8LB	0	424000000
166	2015	6	A07701-8LB	0	1620000000
167	2015	7	A07701-8LB	1	147000000

168	2015	8	A07701-8LB	1	513000000
169	2015	9	A07701-8LB	1	559000000
170	2015	10	A07701-8LB	0	466000000
171	2015	1	A02449-100	0	719000000
172	2015	2	A02449-100	0	931000000
173	2015	3	A02449-100	0	2090000000
174	2015	4	A02449-100	0	2400000000
175	2015	5	A02449-100	1	2290000000
176	2015	6	A02449-100	1	509000000
177	2015	7	A02449-100	1	108000000
178	2015	8	A02449-100	0	434000000
179	2015	9	A02449-100	1	250000000
180	2015	10	A02449-100	0	207000000
181	2015	1	A05214-3LB	1	805000000
182	2015	2	A05214-3LB	0	864000000
183	2015	3	A05214-3LB	0	1210000000
184	2015	4	A05214-3LB	0	1040000000
185	2015	5	A05214-3LB	0	139000000
186	2015	6	A05214-3LB	1	190000000
187	2015	7	A05214-3LB	0	172000000
188	2015	8	A05214-3LB	0	91400000
189	2015	9	A05214-3LB	0	3700000000
190	2015	10	A05214-3LB	0	431000000
191	2015	1	A07781-4LB	0	409000000
192	2015	2	A07781-4LB	1	1740000000
193	2015	3	A07781-4LB	1	192000000
194	2015	4	A07781-4LB	1	205000000
195	2015	5	A07781-4LB	1	231000000
196	2015	6	A07781-4LB	1	1100000000
197	2015	7	A07781-4LB	1	106000000
198	2015	8	A07781-4LB	1	108000000
199	2015	9	A07781-4LB	1	544000000
200	2015	10	A07781-4LB	1	59900000
201	2015	1	463-4	1	749000000
202	2015	2	463-4	1	584000000
203	2015	3	463-4	1	321000000
204	2015	4	463-4	0	1550000000
205	2015	5	463-4	1	3040000000
206	2015	6	463-4	0	137000000
207	2015	7	463-4	0	559000000
208	2015	8	463-4	1	1440000000
209	2015	9	463-4	1	58600000

210	2015	10	463-4	1	63000000
211	2015	1	P2-4	0	122000000
212	2015	2	P2-4	1	167000000
213	2015	3	P2-4	0	251000000
214	2015	4	P2-4	0	2280000000
215	2015	5	P2-4	1	475000000
216	2015	6	P2-4	1	224000000
217	2015	7	P2-4	1	2780000000
218	2015	8	P2-4	1	501000000
219	2015	9	P2-4	1	2210000000
220	2015	10	P2-4	1	602000000

*Lso status detemined based on molecular evaluation of 4 tubers/plant.
