# Supplementary material for: Characterization of host plant resistance to zebra chip disease from species-derived potato genotypes and the identification of new sources of zebra chip resistance
Source: PLoS One. 2017 Aug 23;12(8):e0183283. doi: 10.1371/journal.pone.0183283 (PMC5568414; doi:10.1371/journal.pone.0183283)
Supplement: S2 Table — (PDF) [file pone.0183283.s002.pdf]

ID	Year	Genotype	Mean Fresh Symp.*	Mean Fried Symp.*	Mean Lso Titer*	Log Lso titer
1	2014	463-4	1.5	1.75	107924.93	5.03
2	2014	463-4	0.75	2.25	372358.67	5.57
3	2014	463-4	1.5	1.5	31660.71	4.5
4	2015	463-4	0	1.38	366616.87	5.56
5	2015	463-4	0	0	1494341.43	6.17
6	2015	463-4	0	0	1632261.33	6.21
7	2015	463-4	0.88	2	75863599.1	7.88
8	2015	463-4	0	0.75	21132.97	4.32
9	2015	463-4	0	1	32575.2	4.51
10	2015	463-4	0	1.13	46189.91	4.66
11	2014	A02449-100	1.25	1.75	1129791.47	6.05
12	2014	A02449-100	0.75	2.5	6179298.52	6.79
13	2014	A02449-100	0	0.75	4535809.92	6.66
14	2015	A02449-100	1.5	2.5	149165704	8.17
15	2015	A02449-100	0	1	62105.26	4.79
16	2015	A02449-100	0	1	47193.64	4.67
17	2015	A02449-100	0	1.13	8623214.51	6.94
18	2014	A05214-3LB	1	1	167915.03	5.23
19	2015	A05214-3LB	0	1.38	58445.72	4.77
20	2015	A05214-3LB	1.88	2.75	48293170.95	7.68
21	2014	A05379-211	3	2	79813.65	4.9
22	2014	A05379-211	0	0	6271.74	3.8
23	2014	A05379-211	0	0	1747.92	3.24
24	2014	A05379-211	0	0	1171.14	3.07
25	2014	A07701-6LB	0	2.25	151284.47	5.18
26	2014	A07701-6LB	1.5	1.5	64163.36	4.81
27	2014	A07701-6LB	0	0.5	72299.74	4.86
28	2014	A07701-6LB	0.5	2.25	1301005.51	6.11
29	2014	A07701-6LB	2	2.25	1301005.51	6.11
30	2015	A07701-6LB	0.63	1.5	15114575.59	7.18
31	2015	A07701-6LB	0	1	28769404.1	7.46
32	2014	A07701-8LB	3	2.25	265955.48	5.42
33	2014	A07701-8LB	3	2.75	92856.73	4.97
34	2014	A07701-8LB	3	3	92822.03	4.97
35	2014	A07701-8LB	3	3	116905.61	5.07
36	2015	A07701-8LB	0	1.25	288237.6	5.46
37	2015	A07701-8LB	0	1.25	41038.02	4.61
38	2015	A07701-8LB	0.13	0.88	163594.17	5.21
39	2015	A07701-8LB	0	1.13	40993.38	4.61
40	2015	A07701-8LB	0	1	5678653.69	6.75
41	2014	A07705-4LB	2	3	244597.65	5.39

42	2014	A07705-4LB	2	2.5	69722.75	4.84
43	2014	A07705-4LB	1	1.25	53505.56	4.73
44	2014	A07705-4LB	1.5	2.25	442287.31	5.65
45	2014	A07705-4LB	0.75	3	1108682.32	6.04
46	2014	A07705-4LB	0	0	22967.61	4.36
47	2015	A07705-4LB	0	1.25	18817.45	4.27
51	2014	A07781-4LB	0	1	1076679.27	6.03
52	2014	A07781-4LB	0	0	3472946.33	6.54
53	2014	A07781-4LB	0	1	98129.4	4.99
54	2015	A07781-4LB	0	1	1111775.81	6.05
55	2015	A07781-4LB	0.13	1	150151.48	5.18
56	2015	A07781-4LB	0.13	1.13	74064.22	4.87
57	2015	A07781-4LB	0	0	14299.56	4.16
58	2015	A07781-4LB	0	1	184347.19	5.27
59	2015	A07781-4LB	0	1	249225.54	5.4
60	2015	A07781-4LB	0	1	2786132.68	6.45
61	2015	A07781-4LB	0	1	607962.33	5.78
62	2015	A07781-4LB	0.25	1.13	687194.32	5.84
63	2014	P2-4	3	3	848972.36	5.93
64	2014	P2-4	2.5	2.75	3772132.16	6.58
65	2014	P2-4	2.5	2.5	908321.88	5.96
66	2014	P2-4	2	2.75	1970092.97	6.29
67	2015	P2-4	0	1	69543.03	4.84
68	2015	P2-4	0	1.5	64569.97	4.81
69	2015	P2-4	0.13	1.38	37935.73	4.58
70	2015	P2-4	0	1	26791.85	4.43
71	2015	P2-4	0	1.13	74635.73	4.87
72	2015	P2-4	0.25	2	87163.6	4.94
73	2015	P2-4	0	1.25	71089.42	4.85
74	2014	RB	3	1.5	42927.55	4.63
75	2014	RB	2.5	2	56934.17	4.76
76	2015	RB	0.13	1.25	31723.13	4.5
77	2015	RB	2.25	1.88	14839937.4	7.17
78	2015	RB	0.88	1.5	11798788.72	7.07

*Mean Lso and symptom severities are based on 2 tubers in 2014 and 4 tubers in 2015
